# Circ_0007841 accelerates ovarian cancer development through facilitating MEX3C expression by restraining miR-151-3p activity

**DOI:** 10.18632/aging.202911

**Published:** 2021-04-25

**Authors:** Kejin Huang, Dan Liu, Changlei Su

**Affiliations:** 1 Department of Gynecology, Harbin Medical University Tumor Hospital, Harbin 150001, China; 2 The Seventh Department of Internal Medicine, Harbin Medical University Tumor Hospital, Harbin 150001, China; 3 Department of General Surgery, The Second Hospital of Harbin Medical University, Harbin 150001, China

**Keywords:** ovarian cancer, circ_0007841, miR-151-3p, MEX3C, development

## Abstract

The critical importance of circular RNAs (circRNAs) in human cancers, including ovarian cancer, has been discovered in the recent years. However, the roles of circ_0007841 in ovarian cancer remain unknown. In the current study, it was found that circ_0007841 expression was upregulated in ovarian cancer tissues and cell lines. Upregulation of circ_0007841 in patients with ovarian cancer predicts poor prognosis. Loss-of-function experiments discovered that circ_0007841 knockdown suppressed the proliferation, migration and invasion of ovarian cancer cells *in vitro* and *in vivo*. In terms of mechanism, circ_0007841 worked as a competing endogenous RNA (ceRNA) for miR-151-3p to facilitate MEX3C expression. Restoration of MEX3C level recovered the proliferation, migration and invasion of ovarian cancer cells. In conclusion, this study demonstrated that circ_0007841/miR-151-3p/MEX3C axis exerted important oncogenic functions in ovarian cancer.

## INTRODUCTION

Ovarian cancer (OC) is a very malignant cancer derived from the ovary [1]. Over 20,000 new patients with OC were diagnosed and about 14,000 OC resulted in deaths every year only in USA [2]. Although the approaches for OC treatment have been improved in the past years, the five year survival rate in OC patients remains less than 40% [3]. Thus, illustrating the mechanism of OC development and progression is quite important for developing novel effective therapeutic targets.

Circular RNAs (circRNAs) are another member of noncoding RNAs (ncRNAs), which are characterized by a closed loop structure [4]. CircRNA is resistant to exonuclease and very stable [5]. Hence, circRNAs may be potential biomarkers for diagnosis or prognosis. Recent evidence has demonstrated that circRNAs are extensively expressed in tumor tissues [6]. Aberrant expression of circRNAs may cause tumorigenesis. Moreover, circRNAs participate in multiple biological processes in cancer cells through regulating proliferation, apoptosis or metastasis [7]. For instance, circSEC31A contributes to lung cancer proliferation and invasion through inhibiting miR-376a [8]. Hsa_circ_0004872 is a tumor suppressor to repress gastric cancer cell proliferation and migration [9]. In addition, circSEMA5A overexpression enhances bladder cancer cell proliferation and migration via facilitating ENO1 expression [10]. Therefore, it is essential to investigate the function of OC-associated circRNAs.

Circ_0007841 is a biomarker for diagnosis in multiple myeloma [11]. Recent study indicates that circ_0007841 also affects multiple myeloma progression and drug resistance [12]. Nevertheless, its role in OC is unclear. This current study showed that circ_0007841 was upregulated in OC tissues and is a prognostic biomarker. Functional experiments illustrated that circ_0007841 promoted OC progression *in vitro* and *in vivo* through sponging miR-151-3p to enhance MEX3C expression.

## RESULTS

### Circ_0007841 is highly expressed in OC tissues

**Figure 1:**
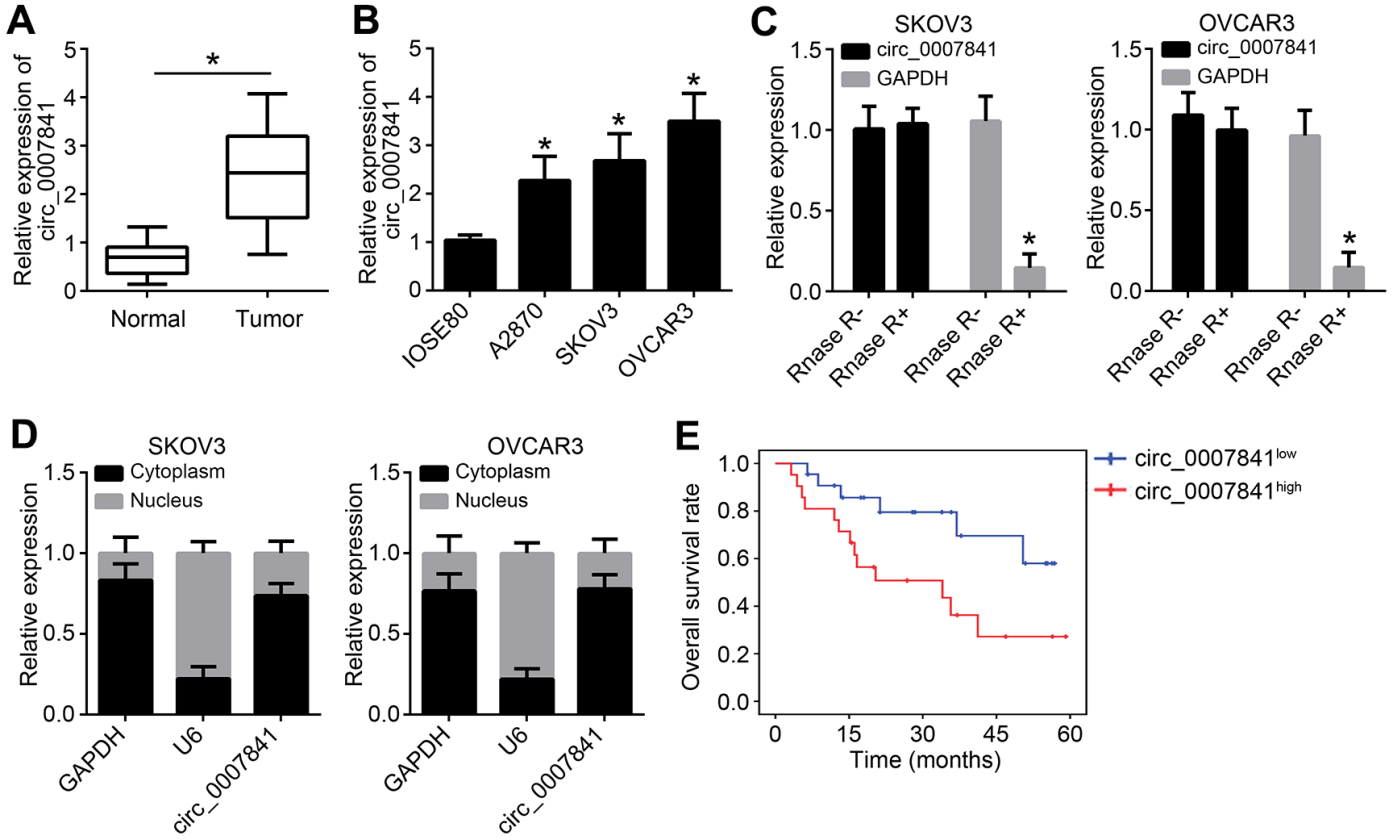
**circ_0007841 is highly expressed in OC tissues.** (**A**) Circ_0007841 expression in OC tissues and adjacent normal tissues was detected by qRT-PCR. (**B**) Circ_0007841 expression in OC cell lines were measured by qRT-PCR. (**C**) Circ_0007841 expression was analyzed after RNase RNA digestion. (**D**) Subcellular distribution of circ_0007841 was analyzed by qRT-PCR. (**E**) Overall survival rate was determined based on circ_0007841 median value in OC tissues. *P<0.05 by Student’s t-test.

The expression of circ_0007841 in OC was firstly detected. Using qRT-PCR, it was found that circ_0007841 level was increased in OC tissues compared to normal tissues (Figure 1A). Circ_0007841 expression was also up-regulated in OC cell lines compared to IOSE80 cells (Figure 1B). To validate circ_0007841 as a circRNA, we treated RNA with RNase R, followed by qRT-PCR. Circ_0007841 was resistant to RNase R digestion (Figure 1C). It is noticed that circ_0007841 was mainly distributed in the cytoplasm of OC cells (Figure 1D). Then, OC samples were divided into two groups based on circ_0007841 median value. We found that circ_0007841 overexpression was associated with a low survival rate (Figure 1E), indicating circ_0007841 may be a prognostic biomarker.

### Circ_0007841 overexpression promotes OC proliferation, migration and invasion

**Figure 2:**
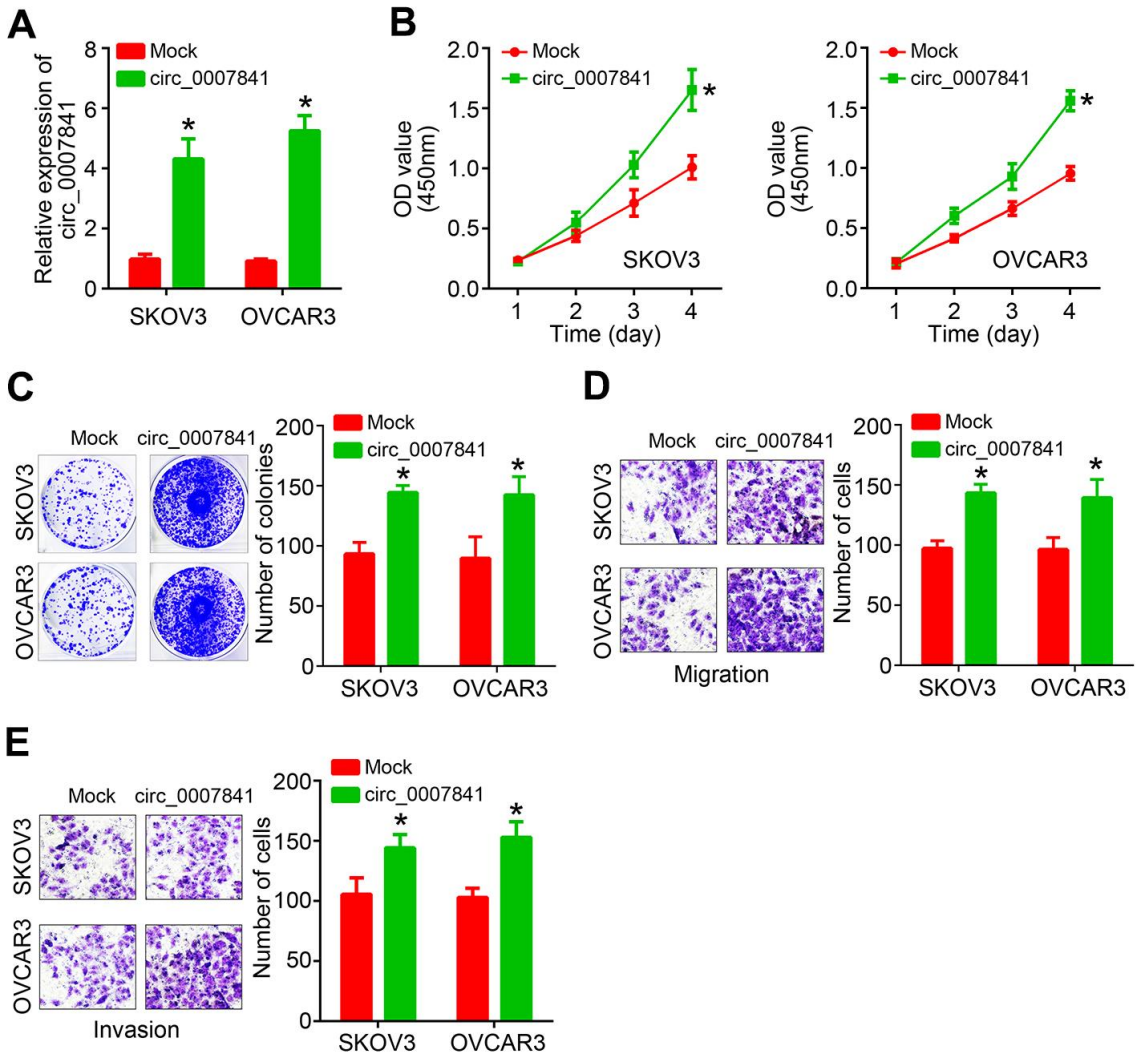
**circ_0007841 overexpression promotes OC proliferation, migration and invasion.** (**A**) Validation of circ_0007841 upregulation in OC cells after transfection. (**B**, **C**) CCK8 and colony formation assays used for cell proliferation analysis. (**D**, **E**) Transwell assays were performed to determine migration and invasion. *P<0.05 by Student’s t-test.

Next, circ_0007841 was ectopically expressed in SKOV3 and OVCAR3 cells (Figure 2A). CCK8 assay showed that circ_0007841 overexpression enhanced the proliferation rate (Figure 2B). Colony formation assay indicated that circ_0007841 upregulation caused more colony formation (Figure 2C). Transwell migration and invasion assays illustrated that circ_0007841 ectopic expression promoted cell migration and invasion (Figure 2D, 2E).

### Circ_0007841 knockdown suppresses malignant biological behaviors

**Figure 3:**
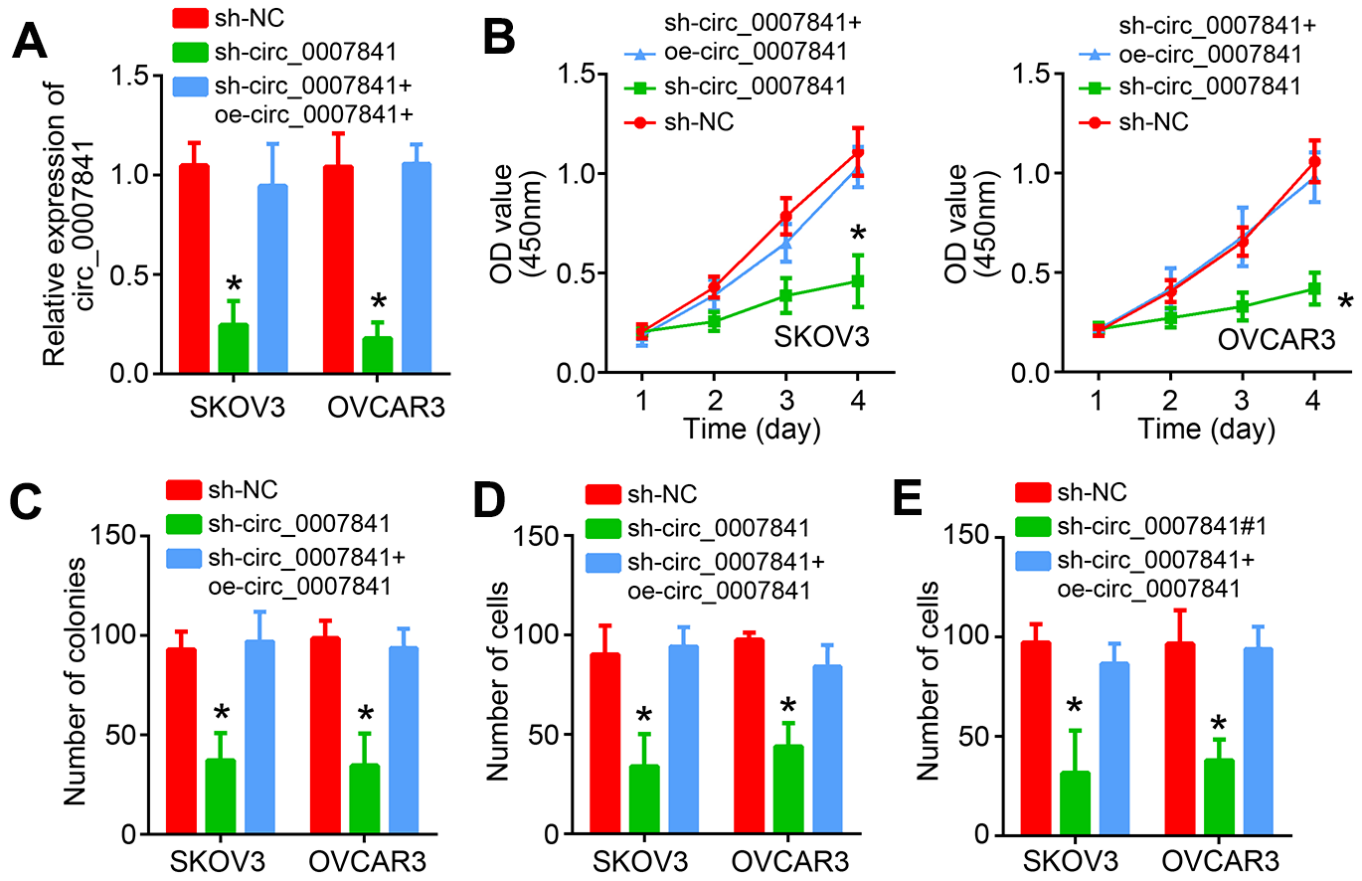
**circ_0007841 knockdown suppresses malignant biological behaviors.** (**A**) Validation of circ_0007841 silencing by qRT-PCR. (**B**, **C**) Cell proliferation was detected by using CCK8 and Transwell assays. (**D**, **E**) Transwell assays were carried out to analyze migration and invasion. *P<0.05 by Student’s t-test.

To further validate the roles of circ_0007841, we knocked it down through shRNA in SKOV3 and OVCAR3 cells (Figure 3A). Similarly, CCK8 and colony formation assays showed that circ_0007841 knockdown inhibited the proliferation rate of OC cells (Figure 3B, 3C). Transwell assays also suggested that circ_0007841 downregulation resulted in less migrated or invaded cells (Figure 3D, 3E). Notably, rescue expression of circ_0007841 abrogated the effects of its silencing (Figure 3B–3E). Thus, circ_0007841 promotes OC progression.

### Circ_0007841 is the ceRNA for miR-151-3p

**Figure 4:**
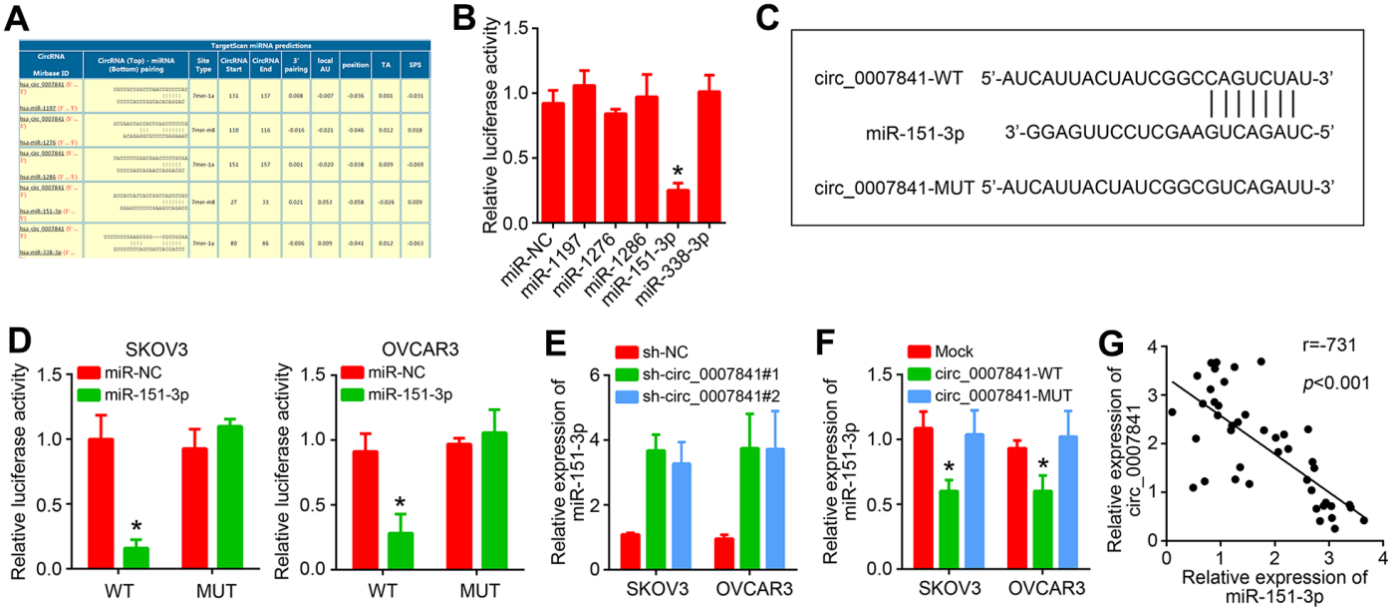
**circ_0007841 is the ceRNA for miR-151-3p.** (**A**) Predicted targets of circ_0007841 by circinteractome. (**B**) Luciferase reporter assay was performed to validate the interaction between circ_0007841 and predicted targets. (**C**) Construction of wild-type (WT) and mutant (MUT) reporter vector. (**D**) Luciferase reporter assay using constructed luciferase reporters. (**E**) qRT-PCR analysis of miR-151-3p expression after circ_0007841 silencing. (**F**) qRT-PCR analysis of miR-151-3p expression after circ_0007841 overexpression. (**G**) Expression correlation between circ_0007841 and miR-151-3p in OC tissues. *P<0.05 by Student’s t-test.

To investigate the molecular mechanism, the potential targets of circ_0007841 were predicted by using circinteractome (https://circinteractome.nia.nih.gov/). The five most potential targets were listed (Figure 4A). Luciferase reporter assay showed that only miR-151-3p mimics inhibited the activity of circ_0007841 reporter (Figure 4B). We then constructed corresponding wide-type and mutant luciferase reporters (Figure 4C). Luciferase reporter assay showed that only circ_0007841-WT reporter activity was suppressed by miR-151-3p mimics (Figure 4D). Besides, miR-151-3p expression was increased after circ_0007841 silencing (Figure 4E). On the contrary, only circ_0007841-WT overexpression repressed miR-151-3p level in OC cells (Figure 4F). qRT-PCR analysis showed that miR-151-3p level was negatively correlated with circ_0007841 in OC tissues (Figure 4G).

### MiR-151-3p targets MEX3C

**Figure 5:**
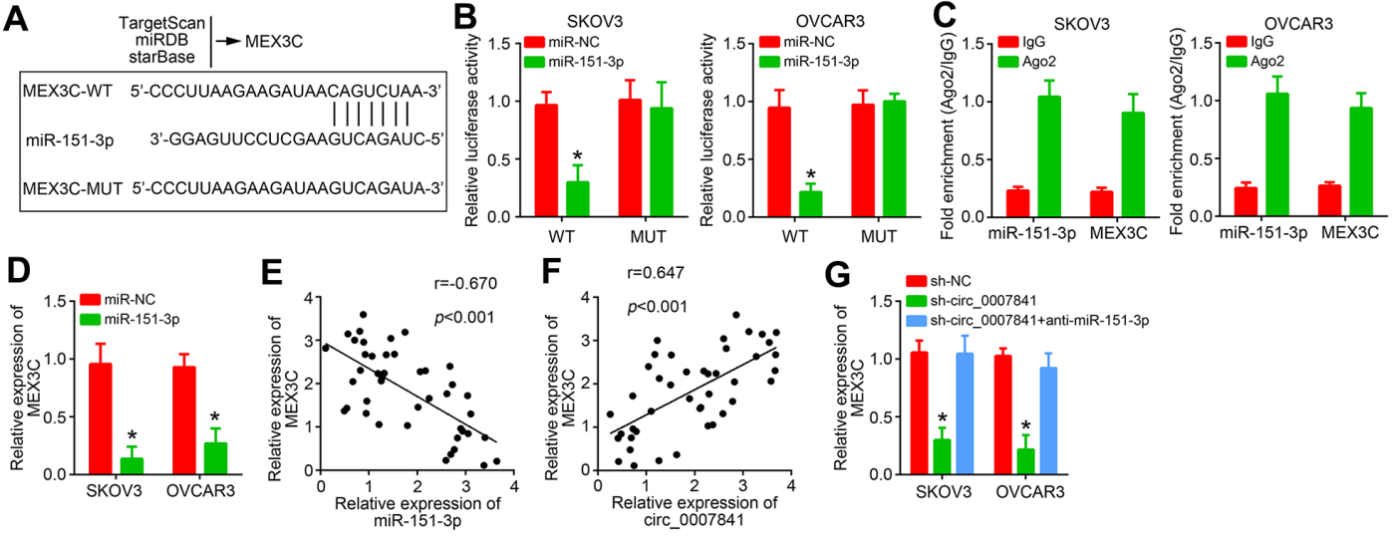
**miR-151-3p targets MEX3C.** (**A**) MEX3C was predicted to be the target of miR-151-3p via TargetSan, miRDB and starBase. (**B**) Luciferase reporter assay. (**C**) RIP assay was performed to validate the interaction between miR-151-3p and MEX3C. (**D**) Relative expression of MEX3C was analyzed by qRT-PCR. (**E**) Expression correlation between MEX3C and miR-151-3p in OC tissues. (**F**) Expression correlation between MEX3C and circ_0007841 in OC tissues. (**G**) Relative expression of MEX3C was analyzed by qRT-PCR after transfection. *P<0.05 by Student’s t-test.

Afterwards, the targets of miR-151-3p were predicted through three online tools and MEX3C was identified (Figure 5A). Similarly, the wide-type and mutant luciferase reporters were constructed (Figure 5A). Luciferase reporter assay indicated that MEX3C-WT reporter activity was suppressed by MEX3C mimics (Figure 5B). RIP assay showed that Ago2 could enrich both MEX3C mRNA and miR-151-3p (Figure 5C). In addition, miR-151-3p mimics inhibited the expression of MEX3C (Figure 5D). We noticed that miR-151-3p level was negatively correlated with MEX3C in OC tissues (Figure 5E). However, MEX3C level was positively correlated with circ_0007841 (Figure 5F). Circ_0007841 knockdown suppressed MEX3C expression whereas miR-151-3p inhibitors reversed it (Figure 5G), indicating that circ_0007841 promotes MEX3C expression via acting as the ceRNA for miR-151-3p.

### Circ_0007841 promotes OC progression through miR-151-3p/MEX3C axis

**Figure 6:**
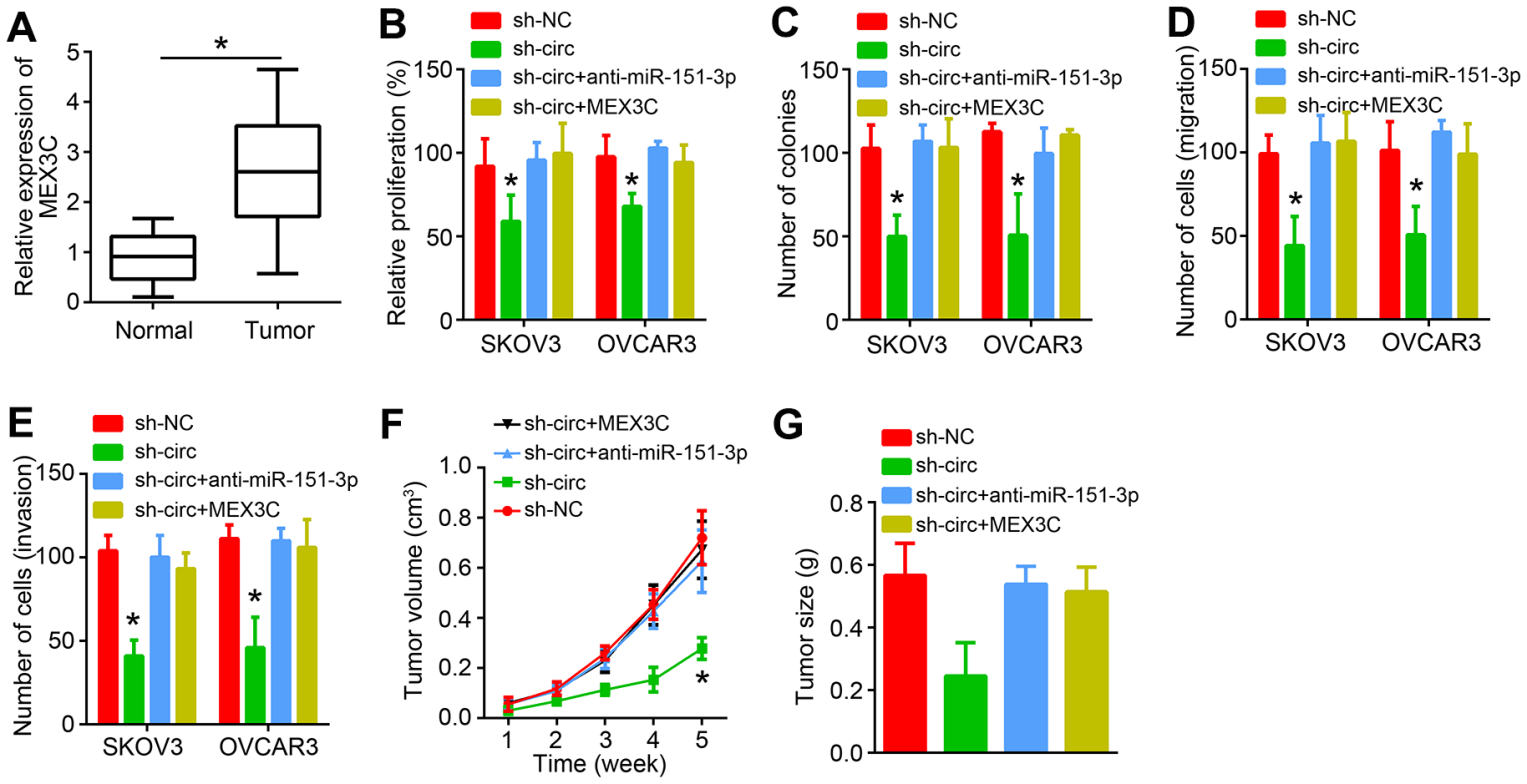
**circ_0007841 promotes OC progression through miR-151-3p/MEX3C axis.** (**A**) MEX3C expression was analyzed in OC tissues by qRT-PCR. (**B**, **C**) CCK8 and colony formation assays were performed to analyze proliferation. (**D**, **E**) Transwell assay was conducted to detect migration and invasion. (**F**) Tumor volumes were measured every one week. (**G**) Tumor size was measured after five weeks. *P<0.05 by Student’s t-test.

It was found that MEX3C was upregulated in OC tissues (Figure 6A). To explore whether circ_0007841 promotes OC progression relying on miR-151-3p/MEX3C axis, rescue assays were carried out. CCK8 and colony formation results showed that miR-151-3p inhibition or MEX3C overexpression rescued the inhibitory effects of circ_0007841 knockdown on proliferation (Figure 6B, 6C). Similarly, Transwell assays showed that miR-151-3p inhibition or MEX3C overexpression rescued the inhibitory effects of circ_0007841 knockdown on migration and invasion (Figure 6D, 6E). To confirm the role of circ_0007841/miR-151-3p/MEX3C axis *in vivo*, animal experiments were performed. Circ_0007841 knockdown suppressed tumor growth while miR-151-3p inhibition or MEX3C overexpression recovered tumor propagation (Figure 6F, 6G).

## DISCUSSION

With the development of high-throughput sequencing technology, an increasing number of circRNAs have been identified. Emerging evidence implies that important involvement of circRNA in human cancers [6]. In this study, we showed that circ_0007841 was highly expressed in OC tissues and associated with poor prognosis. Gain-of-function and loss-of-function experiments demonstrated that circ_0007841 promoted OC cell proliferation, migration and invasion. Animal experiments also illustrated that circ_0007841 promoted OC growth *in vivo*.

In past few years, more and more works have discovered the functions of circRNA in cancer, including OC. For example, circRNA circEXOC6B suppresses OC proliferation and invasion through inhibiting miR-421 [13]. Circular RNA S-7 is upregulated in OC and promotes EMT by restraining miR-641 [14]. In addition, circRNA ITCH is an anti-cancer gene to inhibit OC growth and metastasis via repressing miR-106a [15]. However, in other types of cancer, there is no similar study about circ_0007841 roles. In our study, we discovered its overexpression in OC tissues and implied that circ_0007841 may be a prognostic biomarker. Finally, we demonstrated that circ_0007841 promotes OC growth *in vitro* and *in vivo*. Thus, our data for the first time defined the oncogenic roles of circ_0007841 in OC.

CircRNAs were discovered to exert competing endogenous RNA (ceRNA) for miRNAs in cancer [7]. For instance, circRNA RNF111 is the ceRNA for miR-27b to promote gastric cancer proliferation [16]. Circ-ZNF652 is the ceRNA for miR-29a to enhance liver cancer growth and metastasis [17]. Circ_0007841 has been shown to sponge miR-338 and miR-129 multiple myeloma [12, 18]. In this study, we performed bioinformatics analysis and finally confirmed that circ_0007841 was the ceRNA for miR-151-3p in OC. miR-151-3p is involved in several cancers, such as breast cancer [19], nasopharyngeal carcinoma [20] and colon cancer [21]. Whether miR-151-3p has a similar role in OC is unknown. Our data showed that miR-151-3p expression was inhibited by miR-151-3p. And miR-151-3p inhibition rescued the proliferation, migration and invasion of OC cells, suggesting that it is a tumor suppressor.

Afterwards, bioinformatics was used to search the targets of miR-151-3p. We identified MEX3C. Luciferase reporter assay and RIP assay demonstrated the interaction between miR-151-3p and MEX3C. Besides, we found that circ_0007841 shRNA and miR-151-3p mimics suppressed MEX3C expression. More importantly, MEX3C expression was positively correlated with circ_0007841 and negatively correlated with miR-151-3p in OC tissues. A recent research indicates that MEX3C promotes bladder cancer development by regulating metabolism [22]. Whether MEX3C regulates OC remains unknown. Our study showed that MEX3C was upregulated in OC tissues. MEX3C overexpression promoted OC proliferation, migration and invasion *in vitro* and *in vivo*. Therefore, MEX3C is a new oncogene in OC.

Summarily, we confirmed the significant roles of circ_0007841 in OC malignant progression. Our data demonstrated that circ_0007841 worked as the ceRNA for miR-151-3p to facilitate MEX3C expression and promote OC cell proliferation, migration and invasion. However, the downstream signaling of miR-151-3p/MEX3C is still elusive, which requires investigation in the future.

## MATERIALS AND METHODS

### Tissues collection

43 OC tissues and adjacent normal tissues were collected from The Second Hospital of Harbin Medical University. Tissues were stored in liquid nitrogen and not treated by chemotherapy or radiotherapy before surgery. Association of circ_0007841 level and clinicopathological parameters in OC patients was presented in Table 1. This study was approved by the Ethics Committee of The Second Hospital of Harbin Medical University. All patients provided written informed consents.

**Table 1:** Association of circ_0007841 level and clinicopathological parameters in OC patients.

**Parameters**	**Low (n=22)**	**High (n=21)**	***P*-value**
Age			0.747
≥50 years	16	14	
<50 years	6	7	
Pathological subtype			0.721
Serous	18	16	
Other	4	5	
Tumor size			0.033
≥1 cm	8	15	
<1 cm	14	6	
FIGO stage			0.031
I-II	13	5	
III-IV	9	16	
Pathological grade			0.033
G1-G2	14	6	
G3	8	15	

### Cell culture

Human OC cell lines and normal ovarian epithelial IOSE80 cells were obtained from American Type Culture Collection (ATCC, Rockville, MD, USA). Cells were cultured using DMEM medium supplemented with 10% FBS and 100 U/mL of penicillin/streptomycin. All plasmids and oligonucleotides were purchased from RiboBio (Guangzhou, China). Transfection was carried out through Lipofectamine 3000 reagent following the manufacturer’s protocols.

### RNA extraction and qRT-PCR

Total RNA was extracted using Trizol reagent (Thermo Fisher Scientific) and utilized to synthesize cDNA through igh Capacity cDNA Reverse Transcription Kit (Thermo Fisher Scientific). qPCR was performed using SYBR Premix Ex Taq™ kit (Takara). Relative expression was normalized to GAPDH or U6 and calculated through 2^−ΔΔCt^ method.

### Cell proliferation assays

For Cell Counting Kit 8 (CCK8, Beyotime, China) assay, Cells were seeded into 96-well plates and cultured. At indicated time points, CCK8 solution was added and incubated for 2 hours. OD values at 450 nm were detected through a microplate reader (Bio-Rad Laboratories, Hercules, CA, USA).

For colony formation assay, cells were plated into 6-well plates and cultured for 14 days. Finally, colonies was fixed with 4 % paraformaldehyde, stained with crystal violet and photographed.

### Transwell assay

For migration assay, cells were seeded into the upper part with 200 μl serum-free medium. The lower chamber was filled with 600 μl complete medium. After cultured for 48 h, the cells on the bottom chamber was fixed with 4 % paraformaldehyde, stained with crystal violet and photographed. For invasion assay, the upper chamber was pre-coated with Matrigel and other steps were the same as migration assay.

### Bioinformatics analysis

The correlation between miR-151-3p and MEX3C was predicted by using TargetScan, miRDB and starBase tools.

### Dual-luciferase reporter assay

Reporter vectors were obtained through inserting circ_0007841 or MEX3C sequence into the PGL3 vector (Promega, Madison, WI). For luciferase reporter assay, miR-151-3p and reporter vectors were co-transfected into cells using Lipofectamine 3000. After 48 h, the luciferase activity was measured using the dual-Luciferase Reporter Assay System (Promega, Sunnyvale, CA) based on the manufacturer’s protocols.

### RNA-immunoprecipitation (RIP) assay

RIP assay was performed using Magna RIP RNA-binding protein immunoprecipitation kit (Millipore, MA) according to manufacturer’s instructions. Cell lysates were incubated with IgG (as a negative control) and Ago2 antibody. Then precipitated RNA was analyzed by qPCR.

### Animal experiments

6-week-old BALB/c nude mice were randomly divided into two groups (n=5 for each group). Then SKOV3 cells were inoculated subcutaneously into the flanks of nude mice. Tumor volumes were measured every one week according to the formula: Tumor volume = (length×width^2^)/2. Animal experiments were approved by the Ethics Committee of The Second Hospital of Harbin Medical University.

### Statistical analyses

Results were analyzed by GraphPad Prism 7.0 software (GraphPad, La Jolla, CA, USA) and expressed as the mean ± SD. All experiments were repeated at least three times. P values were analyzed using Student’s t-test for two group comparison or one-way ANOVA for multiple group comparison. Kaplan Meier survival analysis was used for analysis of survival rate and P-value was calculated by the log-rank test. *P*<0.05 was considered as statistically significant.
